# Temporal Team Mental Model and Performance: From the Perspective of Team Process

**DOI:** 10.3389/fpsyg.2021.766268

**Published:** 2021-11-05

**Authors:** Dan Li, Qian Zhang

**Affiliations:** ^1^College of Business and Administration, Huaqiao University, Quanzhou, China; ^2^School of Business, Minnan Normal University, Zhangzhou, China

**Keywords:** temporal team mental model, behavioral integration, task complexity, team performance, team cognition

## Abstract

This paper, based on the survey data of 506 knowledge-based innovation team leaders, employed the regression model and adopted the bootstrap method, to explore the influence of the temporal team mental model on team performance from the perspective of team process. The study results showed that the temporal team mental model has a significant positive predictive effect on team performance; behavioral integration has a mediating effect between temporal team mental model and team performance; task complexity positively moderates the relationship between behavioral integration and team performance and moderates the mediating effects of behavioral integration.

## Introduction

The rapidly changing business environment, technological innovation, and customer needs have brought huge time challenges to team management. The tasks faced by teams in a limited time are more complex and dynamic, showing the features of multitasking, complex dynamics, and time pressure (Kunzelmann and Rigotti, 2021). If an enterprise wants to survive the challenges and develop, it must quickly improve and give full play to its internal flexibility at all levels so that it can achieve effective dynamic coordination among multiple tasks and respond to severe challenges involving time issues at any time like imminent deadlines and often adjusted goals. In addition, since 2019, when the COVID-19 pandemic that has swept the world has brought great challenges to traditional working methods, employees have to abandon traditional face-to-face work in many cases, but communicate and coordinate more through the telephone and the Internet, which brings further great challenges to team management (Yang et al., 2020). In this context, whether knowledge-based innovation teams can better coordinate their work to ensure the completion of tasks becomes more important.

In recent years, more researchers have begun to pay attention to time issues and take the time perspective as a new point for team research (Liu et al., 2017). The fragmentation, ambiguity, uncertainty, and finiteness of time require teams to adopt a “temporal team coordination mechanism” that goes beyond the “clock time concept” in effectively managing team time. While existing studies mainly focus on the explicit temporal team coordination mechanism, relatively few studies center on the implicit temporal team coordination mechanism. In particular, the mechanism and boundary conditions of the team’s implicit temporal coordination mechanism on team performance and other important team outcome variables require further study. Therefore, based on the team process theory, this study intends to take knowledge-based innovation teams as the research object, to explore the implicit temporal team coordination mechanism, namely the mechanism of the temporal team shared mental model on team performance, and to set a frame of reference for the organization of human resource management and team management.

## Theoretical Basis and Research Hypotheses

### Temporal Team Mental Model and Team Performance

Different scholars define the temporal team mental model from different perspectives. For example, Gevers et al. (2006) mentioned that it reflects the extent to which team members believe that they have reached an agreement on time milestones and timetables and have given equal emphasis to time reference points on interdependent tasks. Further, Standifer and Bluedorn (2006) pointed out that the temporal team mental model is used to characterize team members’ consistent understanding of task deadlines, rhythms, and sequences. This paper adopts the view of Zhang and Cen (2014), defining temporal team mental model as the team members’ consistent understanding and representation of the time aspect of the task completion process, which is reflected in the team members’ shared cognition of “task time,” “team standard time,” and “member characteristic time.”

From previous studies, it can be found that there are relatively few studies on the influencing factors of temporal team mental models, mainly focusing on the following aspects. The first is communication, which is considered the most important way of behavioral moderation in a team (Dreyfus et al., 2019). When unconscious team behavior synchronization is impossible, team members may use the explicit team communication process to make their perceptions of time reach a consensus. The temporal team mental model is often considered to be formed in clear communication. The second aspect is time planning and time reminders. Gevers et al. (2009) summarized the antecedent variables of temporal team mental models as time planning, time reminders, and time reflection. They pointed out that the participation of team members in these activities can contribute to the formation of consistent time understanding of the team. The third is time leadership. Liu et al. (2017) conducted an empirical study on project teams in the construction industry and found that the existence of time leadership can have a positive impact on the team’s temporal team model by promoting time reflection.

In terms of the outcome variables of the temporal team mental model, the current related studies mainly focus on the following aspects. The first is on-time completion and team performance. Gevers et al. (2009) pointed out that the dynamic time adjustment between team members based on the high temporal team mental model can increase the fluency of their interactions, thereby improving team performance. Zhu’s (2017) Study also concluded that forming a temporal team mental model is conducive to team introspection, thereby improving the team’s innovation performance. The second aspect is team members’ satisfaction. By developing a temporal team mental model, team members can reach a consensus on task time, member characteristic time, and team standard time. The perception of consensus can create a positive emotion among team members, thus creating mutual trust and satisfaction (Widmann and Mulder, 2020). The third aspect is team learning and team adaptation. Social identity theory suggested that time identity can enhance the identity of team members to the team and other team members, making team members incorporate other members into their internal groups (Arnéguy et al., 2018). Identity can reduce the prejudice and conflict between team members and promote the transfer of knowledge within the team (Liu et al., 2017). Abrantes et al. (2018) conducted four studies with three different samples, concluding that the temporal team mental model promotes impromptu and pre-adaptation of the team and that team learning plays a moderating role in this process.

By developing a temporal team mental model, team members can reach a consensus on task time, member characteristic time, and team standard time. The perception of consensus can create a positive emotion among team members, thus creating mutual trust (Rentsch and Klimoski, 2001). Mcgrath and Kravitz (1982) have long pointed out that the compatibility of an individual with the rhythm of the work environment has a great influence on emotion, cognition, and behavior. Jansen and Kristof-Brown (2005) also found that when individuals’ rhythm is synchronized with the rhythm of their work environment, participants can find great pleasure in synchronizing with their environment. The reason may be that this creates a kind of an orderly and coordinated interaction mode which reduces the sense of uncertainty. The study of Mohammed et al. (2015) showed that the temporal team mental model has a greater impact on team performance in a crisis than in traditional tasks. Santos et al. (2016) found that shared time cognition can further affect team performance through the mediating effect of time conflict and can replace time leadership to reduce time conflict in the team, thus improving team performance. Based on the analysis above, this paper proposes the following hypothesis:

*H1*: The temporal team mental model has a significant positive impact on team performance.

### The Mediating Effect of Behavioral Integration

Integrating the definitions of previous scholars, this study defines behavioral integration as the behavioral process during which team members actively communicate, share information, knowledge, technology, and other resources, and participate in decision-making and teamwork. Behavioral integration is a process of information aggregation. A team with behavioral integration can share information and resources, and make joint decisions. Through the process of behavioral integration, team members are no longer scattered individuals; instead, they are aggregated into a whole to play a role (Hendriks, 2009). The temporal team mental model establishes a basis for team members to understand time and task completion. This moderate team-sharing cognition can positively affect the behavioral integration process by improving team members’ motivation for cooperation and enhancing team members’ perception of consistency. This study mentioned that the temporal team mental model has an impact on team performance through the information aggregation process, namely behavioral integration, based on the following reasons:

Firstly, based on the perspective of social identity, shared temporal team cognition implies team members’ identification and internalization of team goals (Ashforth et al., 2008). Therefore, in order to achieve common goals, team members are naturally more willing to engage in more activities that are beneficial to the team, including actively providing help to team members, sharing their knowledge and resources, and actively coordinating with other members (Kostopoulos et al., 2015). These behaviors can significantly advance the team’s behavioral integration process. Secondly, the establishment of a temporal team mental model can increase team members’ consensus when an event occurs, reduce time ambiguity and time conflicts, and increase team members’ satisfaction (Gevers et al., 2004). With the help of “team identity” for their daily work, it can also provide references for team interaction (Hackel et al., 2018) and a good foundation for the exchange of information and resources between members (Mcgowan et al., 2017). Thirdly, the common understanding of team time and tasks can make members closer psychologically and hold more positive views and attitudes towards others, for which a stronger trust relationship is established between members (Lea et al., 2001). Despite the possibility of disagreements, it is more likely to reduce them through communication. These behaviors contribute to the realization of behavioral integration. Furthermore, from these perspectives, temporal team mental models can actively promote behavioral integration. Based on the analysis above, this study proposes the following hypothesis:

*H2*: The temporal team mental model has a significant positive impact on behavioral integration.

Since the concept of behavioral integration was put forward, scholars have conducted a lot of study on the effects of behavioral integration, mainly focusing on organizational performance (Hu et al., 2017), knowledge sharing (Zhou, 2016), organizational decision-making and strategy (Gu and Wang, 2016), innovative behavior (Zhang and Ke, 2019), and on other outcome variables. Behavioral integration can improve the level of cooperation between team members, give play to teamwork, and effectively aggregate information. The mutual cooperation between team members can effectively integrate and gather individual knowledge, resources, and skills, thus making members have a deeper understanding of the team’s existing knowledge, resources, and other members’ skills—use and improve them, which is conducive to the completion of team tasks (Liang et al., 2015). Effective communication is conducive to integrating various information resources based on which existing product lines can be expanded and enriched, improving the efficiency of existing distribution channels, opening up new market segments, and developing new distribution channels (Liu et al., 2012). Moreover, behavioral integration can help organizations adapt to changes in the external market as soon as possible and help improve organizational performance (Li and Huang, 2013).

In summary, the temporal team mental model can actively affect the team behavioral integration process by improving teamwork motivation, promoting communication and cooperation between members, and building more positive trust relationships. Through the information aggregation process, namely behavioral integration, the team can effectively use and aggregate information, opinions, and resources from different members to truly play the synergetic effect and significantly improve team performance (Liu et al., 2015; Bedwell, 2019). With H1 and H2, this study puts forward the following hypothesis:

*H3*: Behavioral integration plays a mediating role between the temporal team mental model and team performance.

### Moderating Effect of Task Complexity

Innovation tasks in knowledge-based teams often have high complexity (Cheng and Rui, 2021). First of all, the rationality and cognitive level of the task executors are limited, which leads to the fundamental complexity of the tasks. Second, because multiple tasks of different nature may exist at the same time and affect each other, its interaction makes it more complicated to complete the tasks. Third, the effect of task completion may be measured by multiple indicators, and there may even be contradictions between different indicators, making the evaluation of task results more complicated (Peng and Han, 2011).

It is because of the complexity and challenges of the tasks of the innovation team that special attention should be paid to the impact of task complexity. For example, the study of Feng (2012) showed that the task complexity of Research and Development teams could enhance the impact of coordination and detection behavior in cross-border behaviors on innovation performance (Feng, 2012). Further, Liu et al. (2012) conducted a study on Research and Development teams, finding that task complexity can moderate the relationship between behavioral integration and innovation performance. The more complex the task, the stronger the relationship between behavioral integration and innovation performance. When the complexity of the task is low, the team effectiveness depends on the explicit coordination and the abilities of team members to complete the task independently. However, when the team task is complex, it is not enough to rely solely on the cognitive level of the team members. The completion of the task also needs to rely on the combined effect of other factors such as the team’s external resources and team leadership (Noor and Anum, 2021). That is, when the team task environment changes and team members fail to identify and evaluate the environmental changes without making corresponding adjustments, team performance may weaken (Yoon and Choi, 2019). Based on the analysis above, this paper proposes the following hypothesis:

*H4*: Task complexity can moderate the relationship between behavioral integration and team performance; that is, for task complexity, the relationship between behavioral integration and team performance is strong.

Based on the discussion above, this paper further proposes a moderated mediation model; that is, the temporal team mental model indirectly affects team performance through team learning, and this indirect effect depends on task complexity.

**Figure 1:**
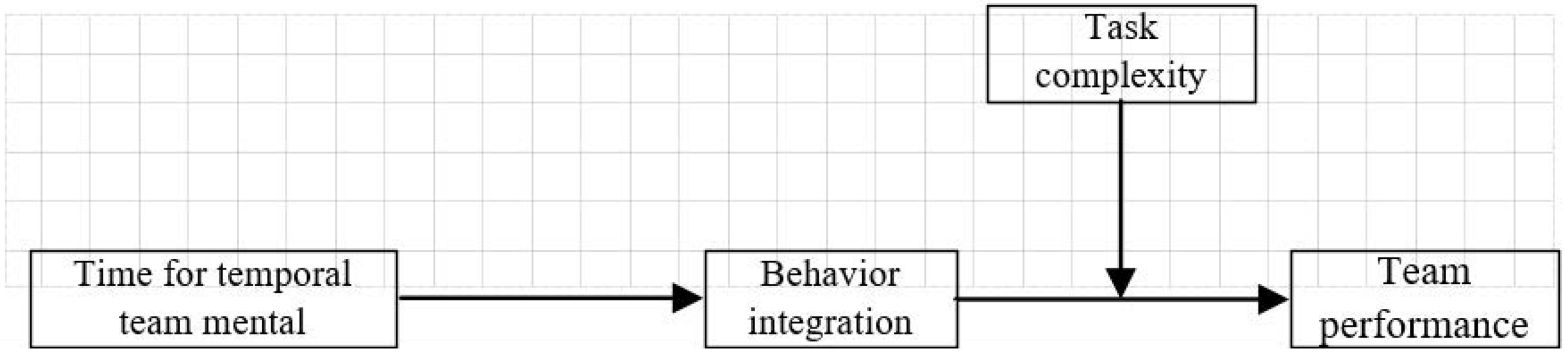
Theoretical model.

*H5*: Task complexity can moderate the mediating effect of behavioral integration in the relationship between the temporal team mental model and team performance. The higher the task complexity, the stronger the effect of behavioral integration in the relationship between the temporal team mental model and team performance. The theoretical model of this study is shown in Figure 1.

## Materials and Methods

### Samples and Procedures

Participants in this study were mainly selected from knowledge-based innovation team leaders in many companies in Xiamen, Chongqing, Shenzhen, Shanghai, Guangzhou, and other regions. They are mainly engaged in innovative work such as software development, product development, and technical support. Questionnaires were designed to collect longitudinal data at two time points (T1, T2) with a time interval of about 3months. The content of T1 involved basic personal information, basic department information, temporal team mental model, behavioral integration, and task complexity. On the other hand, the content of T2 was about team performance. A total of 506 questionnaires were issued. After the option results with obvious regularity and with incomplete answers (missing values) were deleted, a total of 484 valid questionnaires were finally obtained, with a recovery rate of 95%, which meets the statistical requirements.

### Measuring Tools

The questionnaires used in this paper were derived from questionnaires that have been verified to be operational and feasible in authoritative journals. The questionnaires adopted a Likert-type 7-point scale, in which 1 means “completely disagree” and 7 means “completely agree.”

#### Temporal Team Mental Model Scale

Ten items used by Zhang and Cen (2014) were adopted in this study, including task time, team standard time, and member characteristic time.

#### Behavioral Integration Scale

Ten items used by Yao and Sun (2009) were adopted in this study, including three dimensions: decision-making participation, open communication, and teamwork.

#### Task Complexity Scale

Based on the research findings of Stockhomburg (2006) and Joshi et al. (2010), this paper extracted five measurement items from the repeatability, decomposability, and possible paths that may affect task performance.

#### Team Performance Scale

This paper adopted the 4-item scale of Gonzalez-Mule et al. (2014), and the team leader evaluated the teams’ overall task performance completion.

#### Control Variables

Previous studies showed that team size and familiarity among members could affect the interaction and communication within the team (Chen, 2006), so these two were used as control variables. A single-item was adopted to measure the familiarity among team members, that is, “Please evaluate the familiarity among team members” (1=“completely unfamiliar,” 7=“very familiar with each other”).

## Results

### Test of Common Method Bias

This study used Harman’s single factor test for the common method bias. According to this test step, all variables in this paper were included in the factor analysis, and the software SPSS was used for exploratory factor analysis. Among them, the factor with the greatest explanatory power has an Eigen value of 12.140, which explains 23.252% of the total variance. It can be explained that a single factor fails to explain most of the variation; that is, there is no serious common method bias in this paper.

### Reliability and Validity Analysis of Scales

SPSS22.0 and MPLUS8.0 were used to test the reliability and validity of all scales. The results are shown in Table 1.

**Table 1:** Reliability coefficient and validity coefficient of the scale.

Primary variable	Number of entries	Value of Cronbach’s Alpha	Sub-dimensions	Number of entries	Value of Cronbach’s Alpha	Convergent validity
Temporal team mental model	10	0.935	Task time	4	0.943	0.806
			Team standard time	3	0.964	0.899
			Member characteristic time	3	0.956	0.878
Behavioral integration	10	0.948	Decision-making participation	4	0.939	0.795
			Open communication	3	0.944	0.850
			Teamwork	3	0.933	0.824
Task complexity	5	0.879	Task complexity	5	0.879	0.593
Team performance	4	0.940	Team performance	4	0.940	0.798

Cronbach’s Alpha is called the internal consistency coefficient or internal consistency reliability coefficient, indicating the degree to which all items point to agreement. Generally, Cronbach’s Alpha coefficient is above 0.8, which is considered to be highly reliable. It can be found in Table 1 that the Cronbach’s Alpha value of each scale meets the statistical requirements, and the convergent validity of the variables also meets the statistical standards, indicating that all measurement items meet the requirements and the model fits well.

### Correlation Analysis

Kolmogorov-Smirnov tests (K-S test) are non-significant at the 0.05 significance level, so the data can be considered as normal distribution. Pearson correlation was used to calculate the correlation between variables. The mean value, standard deviation, Pearson correlation coefficient, and significance level of each variable are shown in Table 1. It can be found that the correlation coefficients of key variables are basically consistent with the research hypotheses and pass the significance test, such as temporal team mental model and team performance (*r*=0.273, *p*<0.01), temporal team mental model, and behavioral integration (*r*=0.611, *p*<0.01), behavioral integration and team performance (*r*=0.344, *p*<0.01), and task complexity and team performance (*r*=0.248, *p*<0.01), which provides preliminary evidence for the hypotheses testing, as is shown in Table 2.

**Table 2:** Correlation analysis.

S.no.	Variables	Mean	Standard deviation	1	2	3	4	5
1.	Familiarity	4.910	1.398	1				
2.	Temporal team mental model	4.121	1.071	0.691^**^	1			
3.	Behavioral integration	4.460	1.087	0.658^**^	0.611^**^	1		
4.	Team performance	4.283	1.199	0.351^**^	0.273^**^	0.344^**^	1	
5.	Task complexity	4.274	1.178	0.474^**^	0.418^**^	0.408^**^	0.248^**^	1

*n*=484.

** *p*<0.01.

### Hypotheses Testing

To test our hypotheses, we applied the process plug-in developed by Hayes (2013), the deviation-corrected percentile bootstrap method to test the mediating effect of behavioral integration in the relationship between temporal team mental model and team performance. Bootstrap uses repeated sampling technology to extract a certain number of samples from the original samples and then put them back to be extracted again for N times. By repeated extraction for N times (N is generally greater than 1,000 times), which is beneficial to ensure the accuracy of data and the validity of results. The results are as follows:

The temporal team mental model has a significant predictive effect on team performance (*β*=0.306, *p*<0.001), and H1 is verified. Similarly, the temporal team mental model has a significant predictive effect on behavioral integration (*β*=0.718, *p*<0.001), and H2 is verified. After intermediary variables are added, the positive predictive effect of behavioral integration on team performance is also significant (*β*=0.251, *p*<0.001), and the predictive effect of the temporal team mental model on team performance became smaller, indicating that behavioral integration plays a part of the mediating role between the temporal team mental model and team performance, and H3 is verified. In addition, the upper and lower limits of the bootstrap 95% confidence interval of the total effect of the temporal team mental model on team performance and the mediating effect of behavioral integration do not include 0. The direct effect (0.113) and the mediating effect (0.193) account for 36.92 and 63.07% of the total effect (0.306), respectively; H3 is verified, as is shown in Table 3.

**Table 3:** Mediating effect test of behavioral integration.

Dependent variable	Independent variable	Regression coefficient			Fit index		
		*β*	*t*	*p*	*R*	*R* ^2^	*F*
Team Performance	Temporal team mental model	0.306	6.235	<0.001	0.273	0.0746	38.869^***^
Behavioral integration	Temporal team mental model	0.718	16.927	<0.001	0.610	0.372	286.538^***^
Team Performance	Temporal team mental model	0.112	1.864	0.062	0.353	0.124	34.300^***^
	Behavioral integration	0.269	5.252	<0.001			

*β* represents normalized system coefficient; *n*=484.

*** *p*<0.001.

**Figure 2:**
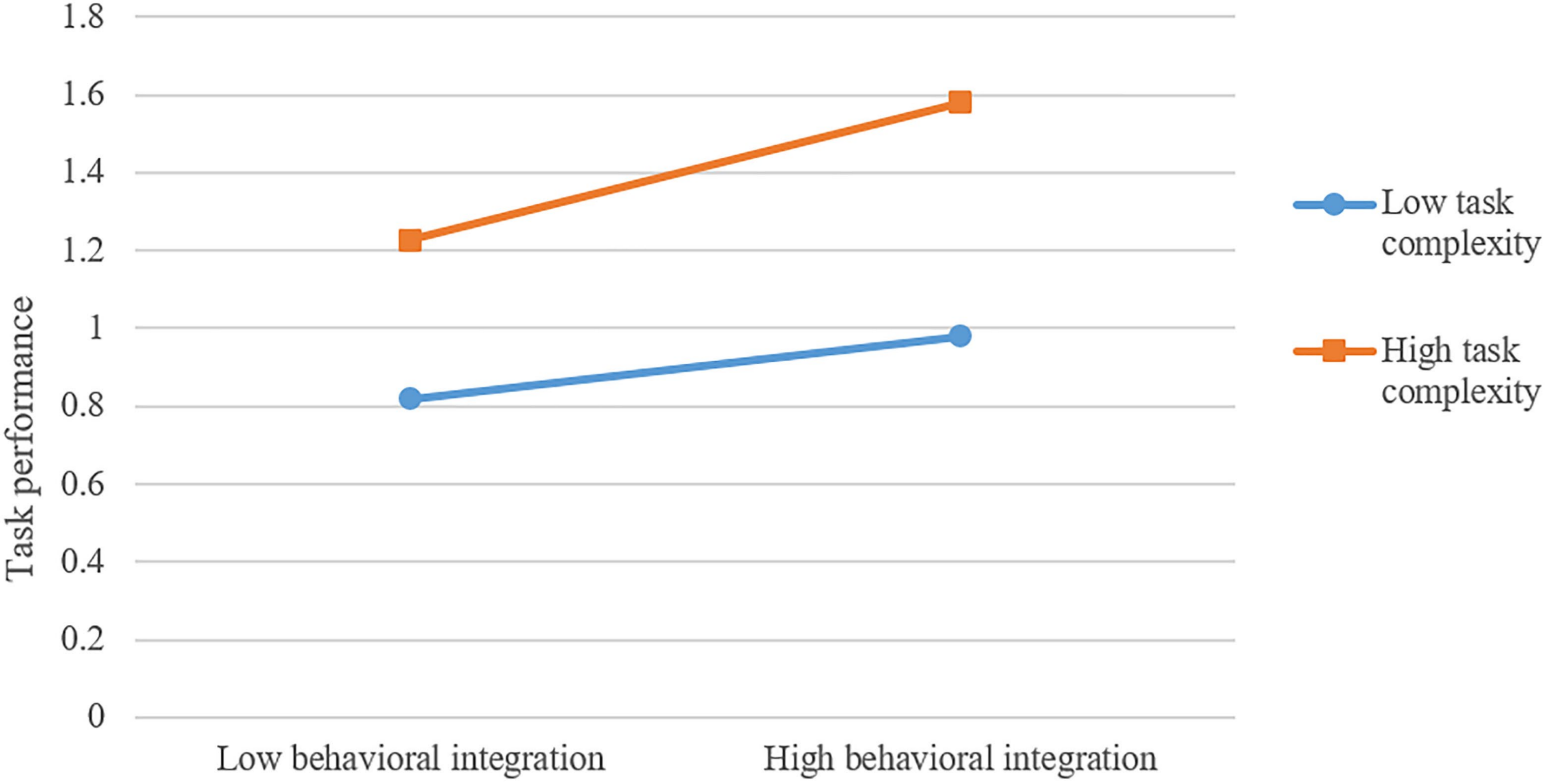
Moderating effect of task complexity.

In order to test the moderating effect of task complexity between behavioral integration and team performance, we applied the SPSS macro compiled by Hayes (2013) to test the moderating effect of task complexity between behavioral integration and team performance. The results in Table 4 show that the interaction items of behavioral integration and task complexity have a significant impact on team performance (*β*=0.093, *p*<0.01). In addition, in the case of high task complexity (M+1SD), behavioral integration has a significant positive predictive effect on team performance (*β*=0.389, *p*<0.001). Meanwhile, in the case of low task complexity (M-1SD), behavioral integration also has a positive predictive effect on team performance (*β*=0.169, *p*<0.05), although its predictive effect is small. Thus, H4 is verified. The slope figure is shown in Figure 2.

**Table 4:** Moderating effect test of task complexity.

	Team performance				
	*β*	Boot standard error	*t*	95% Difference confidence interval	
				Boot CI lower limit	Boot CI upper limit
Behavioral integration (M)	0.679	0.161	4.217^***^	0.362	0.996
Task complexity (W)	0.514	0.155	3.317^***^	0.209	0.818
Interactive item (M^*^W)	0.093	0.036	2.592^**^	0.022	0.164
*R* ^2^	0.144				
*F*	26.992^***^				

*β* represents normalized system coefficient.

** *p*<0.01;

*** *p*<0.001.

In order to verify the moderated mediating effect, we applied the SPSS macro compiled by Hayes (2013) to test the moderated mediating model. The results are shown in Table 5. As seen, the product term of behavioral integration and task complexity has a significant predictive effect on team performance (*β*=−0.094, *p*<0.001), indicating that task complexity can play a moderating role further in the predictive effect of the temporal team mental model on behavioral integration and that the moderated mediating effect model is established. Specifically, when the task complexity is relatively low, the indirect effect of the temporal team mental model integrated into the team performance through behavior is 0.095, which is significant at the 95% confidence interval [0.005, 0.187]; when the task complexity is relatively high, the indirect effect is 0.255, which is significant at the 95% confidence interval [0.158, 0.358]. At the same time, the difference between the two is −0.160, which is significant at the 95% confidence interval [−0.276, −0.041]. H5 is verified.

**Table 5:** Moderated mediating effect model test.

Dependent variable	Independent variable	Regression coefficient				Fit index	
		*β*	*se*	*t*	*p*	*R* ^2^	*F*
Behavioral integration	Temporal team mental model (X)	0.718	0.043	16.927	<0.001	0.373	286.538^***^
Team performance	Temporal team mental model (X)	0.081	0.061	1.322	>0.05	0.147	20.713^***^
	Behavioral integration (M)	0.647	0.162	3.977	<0.001		
	Task complexity (W)	0.503	0.155	3.246	<0.001		
	Interaction item (M^*^W)	0.094	0.036	2.623	<0.05		

*n*=484.

*** *p*<0.001.

## Discussion

Based on the team process theory, this study constructed and tested the theoretical model of team performance of the temporal team mental model–behavioral integration in accordance with the localized management situation in China, focusing on the boundary mechanism of the influence of the temporal team mental model on team performance. It is found that the temporal team mental model has a significant positive impact on team performance; that is, when team members establish a temporal team-shared mental model, it can ensure the common understanding and integrity of team tasks. Behavioral integration plays a part in the mediating role in the relationship between the temporal team mental model and team performance; that is, the temporal team mental model can be used for team performance through behavioral integration. Task complexity can moderate the effect of behavioral integration on team performance, while the mediating role of behavioral integration in the temporal team mental model and team performance can be moderated by task complexity.

### Theoretical Implications

This study has important theoretical contributions to the literature on team cognition and team effectiveness. First, it describes in detail the path of the temporal team mental model affecting team performance. Although some studies have paid some attention to the relationship between shared temporal team cognition identification and team performance (Lea et al., 2001), they do not provide a detailed description of the mechanism between temporal team mental models and team performance. Based on the team process theory, this study conducts an in-depth analysis of the mediating role of behavioral integration between the temporal team mental model and team performance and deepens the understanding of the relationship between the temporal team mental model and team performance. Second, this study explores the application of task complexity as a boundary condition in the study of team implicit time cognition and team effectiveness. Although some scholars have explored the moderating variables between team implicit cognition and team effectiveness, the investigation of task complexity as a boundary condition in the study of team implicit cognition is still limited. This study theoretically clarifies the difference between high and low task complexity in the effect of team time cognition and then responds to the call of previous studies, thus expanding the application of task structure theory in the literature of team implicit time cognition. Third, it appropriately fills in the gap of team effectiveness studies from the collective motivation perspective. Although in the study of individual performance, scholars focus on exploring individual work mobilization, especially the influence of intrinsic motivation on individual performance, there is still a lack of research on how collective motivation affects team performance from the perspective of collective motivation. From the perspective of team cognition, namely collective motivation, this paper deeply explores the impact of collective motivation on team effectiveness and further fills in the gap of team effectiveness research from the perspective of collective motivation.

### Managerial Implications

This study also has an important practical guiding significance. First of all, this study gives full attention and gives full play to the role of the temporal team mental model, an implicit temporal team coordination mechanism, and cultivates a consistent understanding of the time aspect of the team task execution process. In a real team, cross-training, interactive communication, and mutual reminders can be used to establish and improve the temporal team mental model. Second, this paper adopts multiple methods to realize the dynamic cycle management of the temporal team mental model. Managers should spend time discussing the temporal team mental model with their employees to determine whether everyone is “on the same page” and see the “panorama” in the same way. Third, it is necessary to comprehensively use multiple temporal coordination mechanisms to manage team time and push the “time” element from the team’s “background” to the “foreground.” In addition, due to the complexity of creating team tasks, except implicit coordination mechanisms, the auxiliary role of the external coordination mechanism should be considered, such as paying attention to the coordination role of the leadership in the allocation of time and resources and the role of coordination in the synchronization of scheduling and team rhythm (Liu et al., 2021).

## Conclusion

Although this paper has achieved some meaningful results, it still has certain limitations. Further studies are expected to explore the following aspects. First, the temporal team mental model’s antecedent variables and consequence variables need to be further explored. This paper mainly explores the process mechanism of the temporal team mental model affecting team performance. In addition to the team performance involved in this study, future studies can further explore its influence on team creativity, turnover intention, team growth, and other variables and internal mechanism. Furthermore, current studies on the antecedent variables of the temporal team mental model are still relatively limited. In the future, the influence of variables such as member personality differences, team cross-training on it, and the internal mechanism can be further explored. Second, other forms of temporal team coordination mechanisms and their operating mechanisms can be explored. The rapid changes in the external environment and the development of information technology have brought huge challenges to the time management of existing teams. In addition to some well-known temporal team coordination mechanisms (like time leadership and organizational time systems), it is possible that “time adaptation” between team members is also a new type of team time management method. Finally, case studies or simulations can be used to explore further the evolution and dynamics of temporal team coordination mechanisms in different periods. In accordance with the life cycle theory, team structure and team tasks are vary at different stages of development. Therefore, it can be inferred that there may be different coordination mechanisms that dominate the team time in different life cycles of the team. Moreover, a variety of methods can be used in the future to explore the principles of this dynamic process and its dynamic mechanism.

## Data Availability Statement

The original contributions presented in the study are included in the article/supplementary material, further inquiries can be directed to the corresponding author.

## Author Contributions

DL and QZ contributed to conception and design of the study. DL organized the database and wrote the first draft of the manuscript. QZ performed the statistical analysis and wrote sections of the manuscript. All authors contributed to manuscript revision, read, and approved the submitted version.

## Conflict of Interest

The authors declare that the research was conducted in the absence of any commercial or financial relationships that could be construed as a potential conflict of interest.

## Publisher’s Note

All claims expressed in this article are solely those of the authors and do not necessarily represent those of their affiliated organizations, or those of the publisher, the editors and the reviewers. Any product that may be evaluated in this article, or claim that may be made by its manufacturer, is not guaranteed or endorsed by the publisher.
